# Oligomerisation of Synaptobrevin-2 Studied by Native Mass Spectrometry and Chemical Cross-Linking

**DOI:** 10.1007/s13361-018-2000-4

**Published:** 2018-06-12

**Authors:** Sabine Wittig, Caroline Haupt, Waldemar Hoffmann, Susann Kostmann, Kevin Pagel, Carla Schmidt

**Affiliations:** 1 Interdisciplinary Research Center HALOmem, Charles Tanford Protein Center, Institute for Biochemistry and Biotechnology, Martin Luther University Halle-Wittenberg, Kurt-Mothes-Str. 3a, 06120 Halle (Saale), Germany; 2 Institute of Chemistry and Biochemistry – Organic Chemistry, Freie Universität Berlin, Takustr. 3, 14195 Berlin, Germany; 3 Fritz-Haber-Institut der Max-Planck-Gesellschaft, Faradaystr. 4-6, 14195 Berlin, Germany

**Keywords:** Cross-linking, Native mass spectrometry, Synaptobrevin-2, Oligomerisation

## Abstract

Synaptobrevin-2 is a key player in signal transmission in neurons. It forms, together with SNAP25 and Syntaxin-1A, the neuronal soluble *N*-ethylmaleimide-sensitive factor attachment protein receptor (SNARE) complex and mediates exocytosis of synaptic vesicles with the pre-synaptic membrane. While Synaptobrevin-2 is part of a four-helix bundle in this SNARE complex, it is natively unstructured in the absence of lipids or other SNARE proteins. Partially folded segments, presumably SNARE complex formation intermediates, as well as formation of Synaptobrevin-2 dimers and oligomers, were identified in previous studies. Here, we employ three Synaptobrevin-2 variants—the full-length protein Syb(1-116), the soluble, cytosolic variant Syb(1-96) as well as a shorter version Syb(49-96) containing structured segments but omitting a trigger site for SNARE complex formation—to study oligomerisation in the absence of interaction partners or when incorporated into the lipid bilayer of liposomes. Combining native mass spectrometry with chemical cross-linking, we find that the truncated versions show increased oligomerisation. Our findings from both techniques agree well and confirm the presence of oligomers in solution while membrane-bound Synaptobrevin-2 is mostly monomeric. Using ion mobility mass spectrometry, we could further show that lower charge states of Syb(49-96) oligomers, which most likely represent solution structures, follow an isotropic growth curve suggesting that they are intrinsically disordered. From a technical point of view, we show that the combination of native ion mobility mass spectrometry with chemical cross-linking is well-suited for the analysis of protein homo-oligomers.

**Electronic supplementary material:**

The online version of this article (10.1007/s13361-018-2000-4) contains supplementary material, which is available to authorized users.

**Graphical Abstract:**
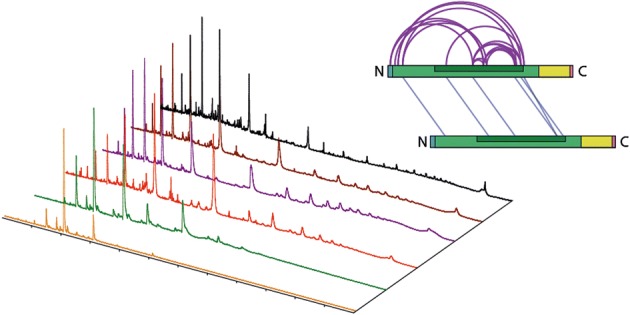
ᅟ

## Introduction

Synaptic vesicles are storage organelles for neurotransmitters in the pre-synaptic nerve terminal. Signal transmission between neurons in the nervous system is mediated by their exocytosis with the pre-synaptic membrane, thereby releasing neurotransmitters into the synaptic cleft which are received by neurotransmitter receptors on the post-synaptic membrane and thus forward the signal [1]. Synaptic vesicles are densely packed with proteins and their major components are identified and well characterised [2, 3]. To date, two quantitative studies provided first insights into the architecture of synaptic vesicles [3] and the neuronal synapse [4] revealing their components, stoichiometries and biophysical properties.

Fusion of the vesicle membrane and the pre-synaptic membrane is performed by so-called SNAREs (i.e. soluble *N*-ethylmaleimide-sensitive factor attachment protein receptor) [5]. During membrane fusion, the mostly unstructured SNARE monomers assemble into the tightly packed SNARE complex [6], a four-helix bundle composed of one α-helix of Synaptobrevin-2 on the vesicle side as well as two SNAP25 and one Syntaxin-1A α-helices on the pre-synaptic membrane [7, 8]. The energy released during complex formation is used to overcome energy barriers for membrane fusion.

**Figure 1:**
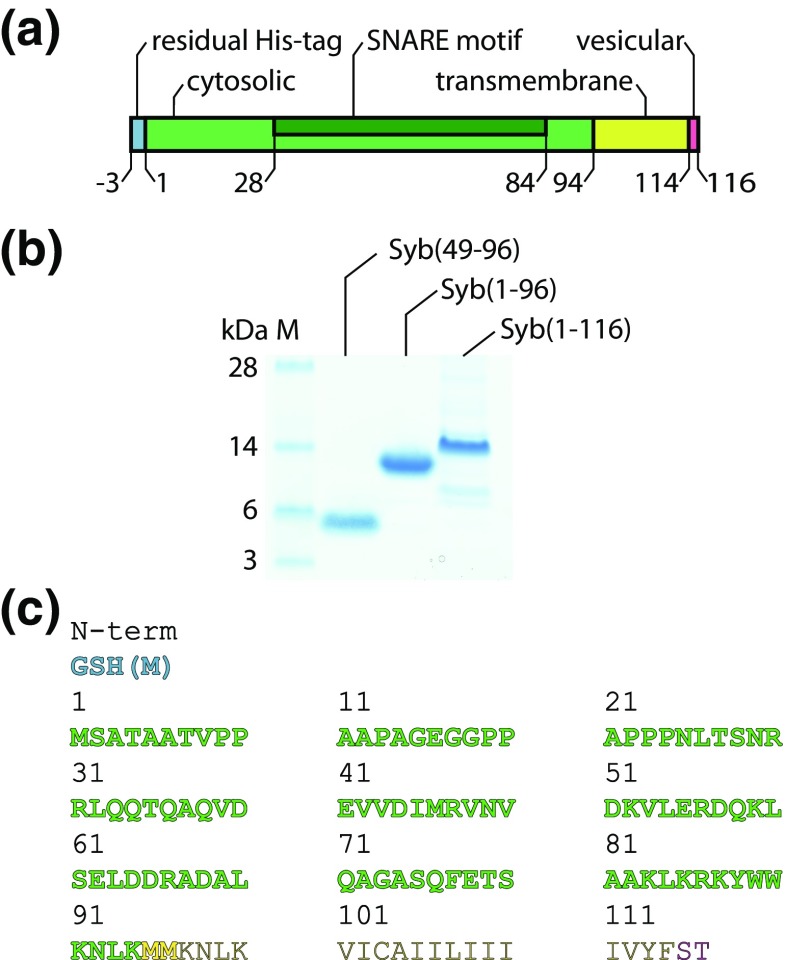
Synaptobrevin-2 variants. **(a)** Bar diagramme showing Synaptobrevin-2 domains. **(b)** Gel electrophoresis of Syb(49-96), Syb(1-96) and Syb(1-116). **(c)** Amino acid sequence of Synaptobrevin-2. Coloured, bold segments were identified by LC-MS/MS. Residual residues of the thrombin cleavage site (N-term) are coloured in blue

The SNARE protein Synaptobrevin-2 is the major component of synaptic vesicles [3]. To gain insights into SNARE complex formation, several structural studies of Synaptobrevin-2 in isolation or in binary complexes were performed. The cytosolic, soluble domain (residues 1-94, Figure 1a) was found to be mostly unstructured [6, 9, 10]. The isolated transmembrane domain (residues 95-114, Figure 1a), on the other hand, is polymorphic containing β-sheet and α-helical fragments depending on the environment [11]. A study on full-length Synaptobrevin-2 showed that the cytosolic domain is unstructured and the transmembrane domain is an inclined α-helix [12]. This model was refined by recent studies showing that the unstructured, cytosolic domain adopts helical structural elements in the presence of the SNARE Syntaxin-1A [6, 9, 13] or in the presence of lipids [14].

Oligomerisation of SNARE proteins has been reported previously [15–19]. In the case of Synaptobrevin-2, this has mostly been described for the transmembrane domain through a conserved amino acid motif in the transmembrane helix [16, 18, 19]. However, dimerization of Synaptobrevin-2 was also questioned [15]. Recently, the oligomerisation of the transmembrane domain was revisited by molecular dynamics simulations of the wild-type and mutated transmembrane domains in a model membrane. This study revealed a high structural stability of Synaptobrevin-2 transmembrane domain oligomers [17].

Here, we employ different Synaptobrevin-2 variants, including the full-length protein, the cytosolic domain and a shorter variant thereof, to study formation of Synaptobrevin-2 oligomers in solution and in the lipid bilayer of proteoliposomes, i.e. in a native-like environment. We combined native mass spectrometry with cross-linking and identified Synaptobrevin-2 oligomers of all variants in solution while full-length Syb(1-116) embedded in the lipid bilayer was mostly monomeric. Importantly, the degree of oligomerisation increased in the truncated versions omitting the transmembrane domain. Using chemical cross-linking, we provide details on the interactions formed within and between the monomeric building blocks. Ion mobility mass spectrometry further revealed the presence of multiple conformations in the short Synaptobrevin-2 variant suggesting isotropic growth for the lower charge states while higher charge states appeared to be unfolded in the gas phase.

## Methods

### Purification of Synaptobrevin-2 Variants

Syb(1-116) and Syb(49-96) plasmids were a gift from Prof. Reinhard Jahn. Syb(1-96) plasmid was cloned from full-length Syb(1-116) according to standard PCR procedures using the following primers:

SB96_Stop_for: 5′-tgg tgg aaa aac ctc aag atg atg taa taa atc atc ttg gga gtg att tgc g-3′ and SB96_Stop_rev: 5′-tta cat cat ctt gag gtt ttt cca cca gta ttt gcg ctt gag ctt ggc -3′ [20].

Synaptobrevin-2 variants (*Rattus norvegicus* sequences, UniProt ID P63045) were purified as described before with minor modifications [21, 22]. Briefly, Syb(1-116), Syb(1-96) and Syb(49-96) were expressed with an N-terminal His-tag in *Escherichia coli* BL21 (DE3) overnight at 22 °C after induction with 0.4 mM IPTG. Proteins were purified via immobilised Ni^2+^ ion metal affinity chromatography (IMAC) after cell disruption using a French press in 20 mM HEPES, pH 7.4, 500 mM NaCl, 0.1 mM TCEP, 1 mM EDTA, and 20 mM imidazole in the presence of protease inhibitor cocktail (Roche). IMAC was performed in the same buffer without EDTA and elution of the proteins occurred at 250 mM imidazole. His-tags were cleaved overnight at 4 °C during dialysis in 20 mM HEPES, pH 7.4, 500 mM NaCl, 0.1 mM TCEP, and 20 mM imidazole using thrombin and removed by reversed IMAC using the same conditions except that the proteins were collected in the flow-through. Finally, Synaptobrevin-2 variants were purified by size exclusion chromatography in 20 mM HEPES, pH 7.4, 150 mM KCl, 0.1 mM TCEP, and 1 mM EDTA, employing 1% (m/v) chaps for full-length Syb(1-116) or omitting the use of detergents for soluble variants. Protein solutions were concentrated using Amicon filtration devices yielding a final protein concentration of 0.156 mM Syb(1-116), 0.696 mM Syb(1-96) and 2.87 mM Syb(49-96).

### Gel Electrophoresis and Western Blotting

Proteins were separated by gel electrophoresis on 4–12% Bis-Tris protein gels using the NuPAGE system (Thermo Fisher Scientific) according to manufacturer’s protocols. SeeBlue Plus2 Pre-stained Protein Standard (Thermo Fisher Scientific) was used as the molecular weight marker. Protein gels were stained with Coomassie using InstantBlue Protein Stain (Expedeon). For western blotting, proteins were transferred to a nitrocellulose membrane (Carl Roth) for 2 h at 50 mA. The membrane was washed with phosphate-buffered saline (PBS)/0.02% Tween-20 and incubated with anti-Synaptobrevin-2 clone 69.1 (SynapticSystems) (1:10,000) or anti-VAMP1/2/3 (SynapticSystems) (1:5000) overnight. The membrane was then incubated with anti-mouse (for anti-Synaptobrevin-2 clone 69.1) or anti-rabbit (for anti-VAMP1/2/3) secondary antibody (both 1:100,000 in PBS/0.02% (*v*/*v*) Tween-20/1% (*w*/*v*) BSA) and the blot was developed with Pierce ECL Western Blotting Substrate (Thermo Fisher Scientific).

### In-Gel and In-Solution Digestion

Proteins were digested in-gel using trypsin or chymotrypsin as described [23] or in-solution using RapiGest surfactant (Waters) according to manufacturer’s protocols. Briefly, protein bands were excised from the gel or proteins in solution were precipitated with ethanol. Disulfide bonds were then reduced with dithiothreitol and free cysteine residues were alkylated using iodoacetamide. Proteins were digested overnight with trypsin or chymotrypsin at 37 or 25 °C, respectively. Generated peptides were dried in a vacuum centrifuge and stored at − 20 °C until use.

### LC-MS/MS

For LC-MS/MS analysis, tryptic peptides were separated by nano-flow reversed-phase liquid chromatography (DionexUltiMate 3000 RSLCnano System, Thermo Scientific; mobile phase A, 0.1% (*v*/*v*) formic acid (FA); mobile phase B, 80% (*v*/*v*) acetonitrile (ACN)/0.1% (*v*/*v*) FA) coupled with a Q Exactive Plus Hybrid Quadrupole-Orbitrap mass spectrometer (Thermo Scientific). The peptides were loaded onto a trap column (μ-Precolumn C18 PepMap 100, C18, 300 μm I.D., particle size 5 μm; Thermo Scientific) and separated with a flow rate of 300 nL/min on an analytical C18 capillary column (50 cm, HPLC column Acclaim® PepMap 100, C18, 75 μm I.D., particle size 3 μm; Thermo Scientific), with a gradient of 4–90% (*v*/*v*) mobile phase B over 69 min for in-gel digested peptides and over 95 min for in-solution digested peptides. Peptides were directly eluted into the mass spectrometer.

Typical mass spectrometric conditions were spray voltage of 2.8 kV; capillary temperature of 275 °C and a normalised collision energy of 30%. The Q Exactive Plus mass spectrometer was operated in data-dependent mode. Survey full scan MS spectra were acquired in the Orbitrap (*m*/*z* 350–1600) with a resolution of 70,000 and an automatic gain control (AGC) target at 3e6. The 20 most intense ions were selected for HCD MS/MS fragmentation in the HCD cell at an AGC target of 1e5. Detection in the HCD cell of previously selected ions was dynamically excluded for 30 s. Singly charged ions as well as ions with unrecognised charge states were also excluded. For cross-linking analysis, doubly charged ions were also excluded. Internal calibration of the Orbitrap was performed using the lock mass option (lock mass *m*/*z* 445.120025 [24]).

### Database Search for Protein Identification

Raw data were searched against a minimised database containing sequences of Synaptobrevin-2 variants using MaxQuant software [25]. The mass accuracy filter was 20 ppm for precursor ions and 20 ppm for MS/MS fragment ions. Peptides were defined to be tryptic or chymotryptic with maximal two missed cleavage sites. Carbamidomethylation of cysteines and oxidation of methionine residues were allowed as variable modifications.

### Native Mass Spectrometry

The purification buffer of Synaptobrevin-2 variants was exchanged to 200 mM ammonium acetate, pH 7.0 using Micro Bio-Spin 6 gel filtration columns (Bio Rad). Detergent was removed from full-length Synaptobrevin-2 using a PD10 column (GE Healthcare; 2.1-mL column volume) packed with Sephadex G25. Intact protein complexes were then analysed on a Q-ToF Ultima modified for transmission of high masses [26]. For this, 2–3 μL of the protein sample was loaded into gold-coated glass capillaries prepared in-house [27]. Typical mass spectrometric conditions were capillary voltage, 1.5–1.7 kV; cone voltage, 80 V; RF lens voltage, 80 V; collision energy, 40–100 V. Mass spectra were processed using MassLynx 4.0 and analysed using *Mass*ign [28] software.

### Chemical Cross-Linking

Varying amounts of Synaptobrevin-2 variants ranging from 10 to 650 μM were cross-linked with varying concentrations of bis(sulfosuccinimidyl)suberate (BS3) cross-linker (80 up to 350 μM) for 1 h at 25 °C and 350 rpm in a thermomixer. For gel electrophoresis and western blotting, lower protein and BS3 concentrations were used to avoid aggregation on the gel (see figure legends for details). Cross-linked proteins were digested in-gel or in-solution with trypsin (see above).

For enrichment of cross-links, the peptide mixture was re-dissolved in 30% (*v*/*v*) ACN, 0.1% (*v*/*v*) trifluoric acid. Linear peptides and cross-linked dipeptides were separated by size exclusion chromatography on a Superdex peptide column 10/300 GL (GE healthcare) at a flow rate of 50 μL/min. Fractions containing mostly cross-linked dipeptides were dried in a vacuum centrifuge and re-dissolved in 2% (*v*/*v*) ACN, 0.1% (*v*/*v*) FA for LC-MS/MS analysis (see above).

For cross-link analysis, raw data were converted into mgf format using pXtract software tool (http://pfind.ict.ac.cn/software.html). Potential cross-links were identified by database search against a minimised database using pLink search engine [29]. The following search parameters were applied: fragmentation, HCD; enzyme, trypsin; variable modifications, oxidation (methionine) and carbamidomethylation (cysteine); cross-linker, BS3. Mass spectra of potential cross-links were manually checked for spectral quality. Cross-links were visualised in bar diagrams using xVis software tool [30].

### Reconstitution of Full-Length Synaptobrevin-2 into Liposomes

Proteoliposomes were prepared by co-micellisation [31]. 1,2-Dioleoyl-sn-glycero-3-phosphocholine (DOPC), 1,2-dioleoyl-sn-glycero-3-phosphoethanolamine (DOPE), 1,2-dioleoyl-sn-glycero-3-phosphoserine (DOPS) and cholesterol (Avanti Polar Lipids) were mixed in chloroform/methanol (2:1 (*v*/*v*)) at a molar ratio of 5:2:2:1 and dried using a rotary evaporator [21]. The dried lipid film was hydrated in 20 mM HEPES, pH 7.4, 150 mM KCl, 0.1 mM TCEP, and 5% (*w*/*v*) sodium cholate yielding multilamellar vesicles with a total lipid concentration of 15 mM. Syb(1-116) in 1% chaps was added at a lipid to protein ratio of 300:1 (*n*/*n*) and incubated for 1 h at 25 °C in a rotary evaporator without evaporation. Detergent removal and spontaneous proteoliposome formation was achieved by size exclusion chromatography using a self-packed Sephadex G25 Superfine column (5/100 mm, bed volume 2 mL) on an Äkta Pure system (GE Healthcare) equilibrated in 20 mM HEPES, pH 7.4, 150 mM KCl, and 0.1 mM TCEP. Proteoliposome-containing fractions were pooled and subjected to a liposome recruitment assay (‘liposome flotation assay’) based on sucrose density gradient centrifugation (sucrose gradient 0 M sucrose (top) – 1.2 M sucrose (bottom)) [32]. The size distribution of the reconstituted liposomes was determined by dynamic light scattering using a Zetasizer Nano (Malvern Instruments).

### Ion Mobility Mass Spectrometry

Collision cross-sections (CCSs) were measured on a home-built drift-tube instrument using helium buffer gas [33]. Ions were generated using a nano-electrospray ionisation (nESI) source and subsequently stored in an hourglass-shaped entrance funnel. They were further transferred by 150-μs-long pulses and an injection voltage of 30 V (soft conditions) into the ion mobility cell, where they travel under the influence of a weak electric field (~ 15 V/cm) through helium buffer gas (~ 5 mbar). During their migration, extended ions collide more frequently with the buffer gas and therefore are retained. The result is a separation of species with identical *m*/*z* ratio but different sizes and shapes. After releasing the ion mobility cell, ions were mass selected using a quadrupole mass filter and their arrival time distributions (ATDs) were recorded by measuring the time-dependent ion current of the *m*/*z*-selected species after release of the ion trap. Arrival time distributions were recorded at five drift voltages (1200–1000 V) and fitted by multiple Gaussian functions. The centre of each Gaussian corresponds to the drift time of a single species and is further converted to a CCS by using the Mason-Schamp equation [34].

## Results

### Purification and Mass Spectrometry of Full-Length Synaptobrevin-2

To study oligomerisation of Synaptobrevin-2, we expressed the full-length protein (i.e. Syb(1-116)) in *E. coli* followed by affinity purification through an N-terminal His-tag (see ‘Methods’). Gel electrophoresis and LC-MS/MS of peptides obtained from in-gel digestion confirmed the presence of the full-length protein (Figure 1). Using both trypsin and chymotrypsin during in-gel digestion covered 75% of the Synaptobrevin-2 sequence and allowed the identification of the complete cytoplasmic domain (residues 2-94) as well as residual N-terminal residues of the thrombin cleavage site (Figure 1c).

**Figure 2:**
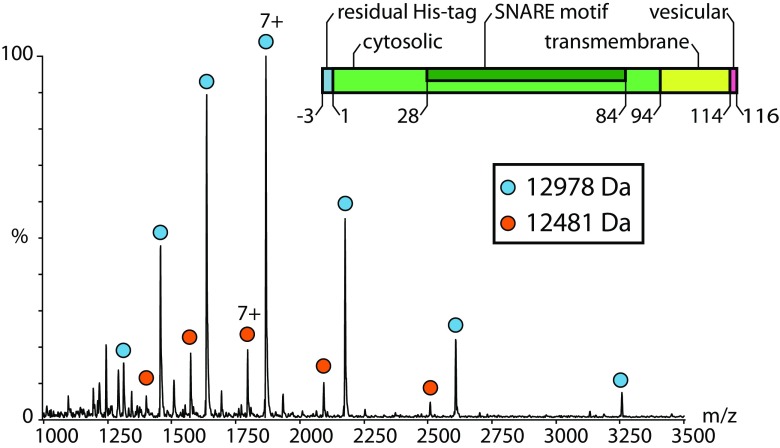
Native mass spectrometry of Syb(1-116). The mass spectrum of intact Syb(1-116) shows one charge state series corresponding to intact Syb(1-116) (blue circles; 12,978 Da). A second charge state series (orange circles) corresponding to smaller mass (12,481 Da) was also observed

Next, we studied Syb(1-116) employing a mass spectrometer modified for transmission of high masses [26]. Although chaps detergent is well-suited for maintaining the native state of proteins, it is not amongst the mass spectrometry-compatible detergents. Consequently, analysis of Synaptobrevin-2 purified in chaps at varying collision energies only revealed detergent clusters overlapping with the protein signals (not shown). As the aggregation number of chaps is very low and the transmembrane domain of Synaptobrevin-2 is rather small, we removed excessive detergent and separated ‘empty’ micelles from the protein-detergent complex by size exclusion chromatography to allow mass spectrometric analysis in minimal amount of detergent. Indeed, a mass spectrum of Syb(1-116) was obtained at moderate collision energy (Figure 2). This mass spectrum showed one major charge state series with a mass of 12,978 Da corresponding to intact Syb(1-116) including residual residues of the thrombin cleavage site (‘Methods’ and Figure 1). Another low abundant peak series with the same charge state distribution but corresponding to a mass of 12,481 Da suggests degradation of the protein (Figure 2 and Table S1). However, following this approach, we did not observe Synaptobrevin-2 oligomers.

### Cross-Linking Full-Length Synaptobrevin-2

**Figure 3:**
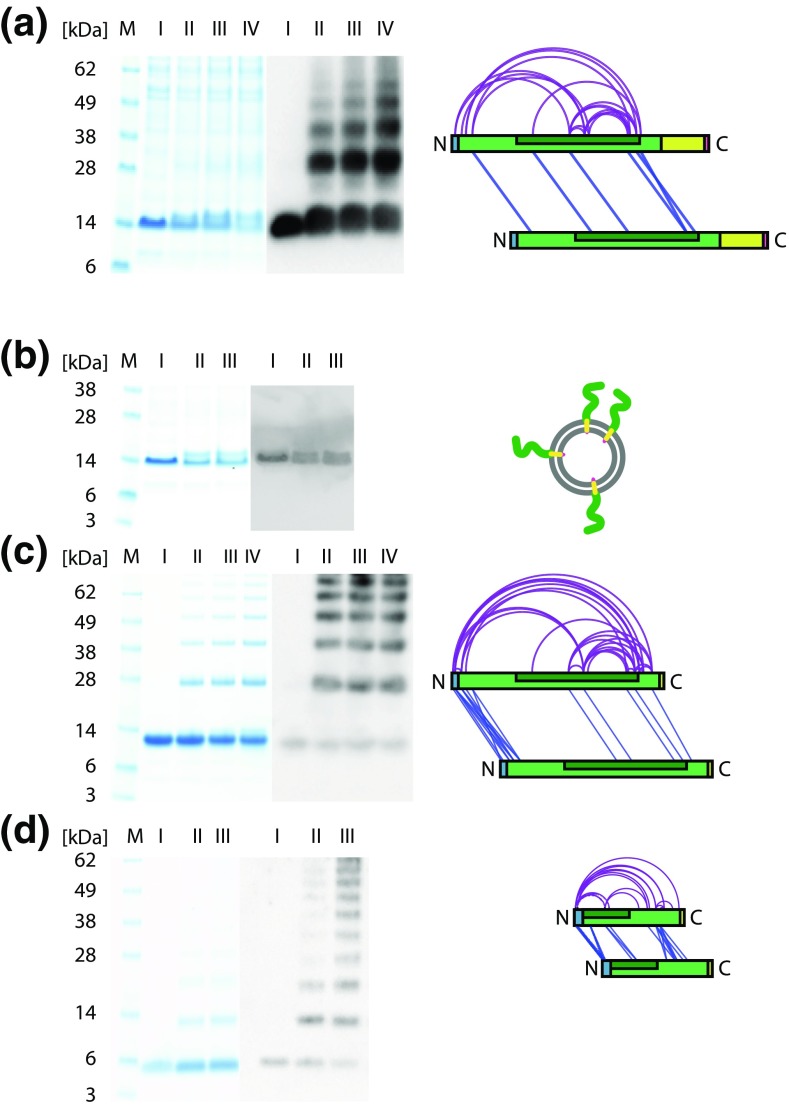
Cross-linking of Synaptobrevin-2 variants. Coomassie-stained gels (lhs) and western blots (middle) are shown. Low BS3 concentrations were used to avoid precipitation of the proteins on top of the gel. Interaction networks (rhs) are shown for all Synaptobrevin-2 variants (**a**, **c**, **d**) and represent binary protein interactions. Proteoliposomes are represented as cartoon (**b**). Western blots were produced using anti-Synaptobrevin-2 clone 69.1 for full-length Synaptobrevin-2 (**a**, **b**) and anti-VAMP1/2/3 for truncated variants (**c**, **d**). **(a)** 100 μM of full-length Syb(1-116) (I) was cross-linked with 83 μM (II), 116 μM (III) and 166 μM (IV) BS3. Oligomers up to hexamers were observed. Higher oligomers could not be resolved by gel electrophoresis. **(b)** 14 μM of full-length Syb(1-116) (I) was cross-linked with 140 μM (II) and 280 μM (III) BS3. No oligomers were observed. **(c)** 200 μM of Syb(1-96) (I) was cross-linked with 180 μM (II), 250 μM (III) and 350 μM (IV) BS3. Oligomers up to hexamers were observed. Higher oligomers could not be resolved by gel electrophoresis. **(d)** 650 μM of Syb(49-96) (I) was cross-linked with 180 μM (II) and 250 μM (III) BS3. Oligomers up to dodecamers were observed

To capture potential low abundant Synaptobrevin-2 oligomers, we performed chemical cross-linking of the full-length protein. As aggregation of Synaptobrevin-2 at higher concentration was controversially discussed [6, 9], we first incubated 10 μM Syb(1-116) with varying amounts of BS3 cross-linker (see ‘Methods’). BS3 covalently links primary amines of lysine side chains or the proteins N-termini and shows some reactivity towards hydroxyl groups of serine, threonine and tyrosine residues. The detergent micelle at the membrane domain should allow access to the reactive residues as shown before [35]. Indeed, gel electrophoresis of the cross-linked protein showed formation of Synaptobrevin-2 dimers when compared with the non-cross-linked protein (Figure S1). Due to higher mass contaminating proteins caught during purification, we could not identify higher oligomers in the Coomassie-stained gel. We therefore used sensitive western blotting to specifically visualise covalently linked Synaptobrevin-2 oligomers. As the intensity of single Synaptobrevin-2 oligomers in a 10-μM solution is low and close to the detection limit of Coomassie-staining we also performed cross-linking at higher Synaptobrevin-2 concentrations and achieved similar results with higher intensities (Figure 3a). In this way, we could show formation of Synaptobrevin-2 oligomers up to pentamers (Figure S1 and Figure 3a).

For identification of cross-linked residues, we then incubated full-length Syb(1-116) with BS3 and digested the covalently linked proteins with trypsin. We then enriched cross-linked dipeptides by size exclusion chromatography and subsequently analysed the fractions containing cross-linked peptides by LC-MS/MS (‘Methods’). Potential cross-linked peptides were identified by database searching using pLink software [29] followed by manual validation of the mass spectra. As described earlier [36], we also considered covalent linkage between lysine and serine, threonine or tyrosine residues in addition to lysine-lysine cross-links. Following this strategy, we identified 23 cross-links in full-length Syb(1-116) (Table S2). Eleven of these cross-links include covalently linked serine and threonine residues. We also identified six cross-links which could unambiguously be assigned to inter-protein interactions, identified by the same or overlapping peptide sequences which can only originate from two copies of Syb(1-116). An example spectrum of the cross-linked dipeptide VNVD[^52^K]VLER - VNVD[^52^K]VLER is shown in Figure S2.

Protein interactions within Syb(1-116) were then visualised in a network plot (Figure 3a). The majority of interactions could not unambiguously be assigned to inter- or intra-protein interactions and most likely corresponds to intra-protein interactions. Cross-links which originate from two Syb(1-116) copies were mostly located at the N-terminus and in the middle region of the protein, i.e. the SNARE motif. No interactions were observed C-terminal of the transmembrane domain although BS3-reactive residues (C-terminal Ser115 and Thr116) are available and the detergent micelle should allow access of the cross-linker as shown in previous studies [35].

### Employing Synaptobrevin-2 Proteoliposomes to Study Oligomerisation in a Native-Like Environment

In previous studies, oligomerisation of Synaptobrevin-2 was mostly described to be driven by the transmembrane domain [16–19]. We therefore incorporated full-length Syb(1-116) into the lipid bilayer of liposomes (see ‘Methods’ for details) and thus mimicked a native-like environment. We employed liposomes containing DOPC:DOPE:DOPS:cholesterol in a 5:2:2:1 molar ratio as liposomes of this composition were successfully applied previously to study SNARE proteins and membrane fusion events [37]. Protein incorporation into the liposomal lipid bilayer was verified by liposome flotation (Figure S3). Indeed, Syb(1-116) was only identified in the upper, low-density fractions of the sucrose gradient confirming its presence in the floating liposome fraction. (Note that solubilised protein not incorporated into the liposomes would be found in the bottom fractions at higher sucrose density.) The size distribution of these proteoliposomes was then analysed using dynamic light scattering showing one homogeneous population of approximately 24-nm-sized proteoliposomes (Figure S4).

Having Synaptobrevin-2 proteoliposomes successfully reconstituted, we incubated them with BS3 cross-linker and studied formation of cross-linked oligomers by gel electrophoresis and western blotting to visualise low abundant oligomers (see above). Both, the Coomassie-stained gel and the western blot only revealed monomeric Syb(1-116). However, the cross-linked monomer showed two protein bands after cross-linking suggesting that two (or even several) conformations could be captured by formation of intra-molecular cross-links and separated by gel electrophoresis.

These experiments suggest that Synaptobrevin-2 is mostly monomeric in proteoliposomes presumably due to separation of the protein in the lipid bilayer. This agrees well with a previous study showing that Synaptobrevin-2 does not form large homo-oligomeric clusters in liposomes of similar composition (PC:PE:PS:phosphatidylinositol:cholesterol 5:2:1:1:1) [38].

### Oligomerisation of the Cytosolic Domain Syb(1-96)

In this study, we did not observe protein interactions in the transmembrane region and found that reconstitution of the full-length protein into the lipid bilayer of liposomes does not promote oligomerisation. However, we identified numerous cross-links in the cytosolic domain (see above, Figure 3a) of the full-length protein in solution. We therefore investigated the cytosolic domain in detail and generated a Synaptobrevin-2 variant omitting the transmembrane domain (i.e. Syb(1-96)). Again, we first identified the protein by LC-MS/MS and, using trypsin and chymotrypsin (see ‘Methods’ for details), covered 98% of the cytosolic domain (Figure 1).

**Figure 4:**
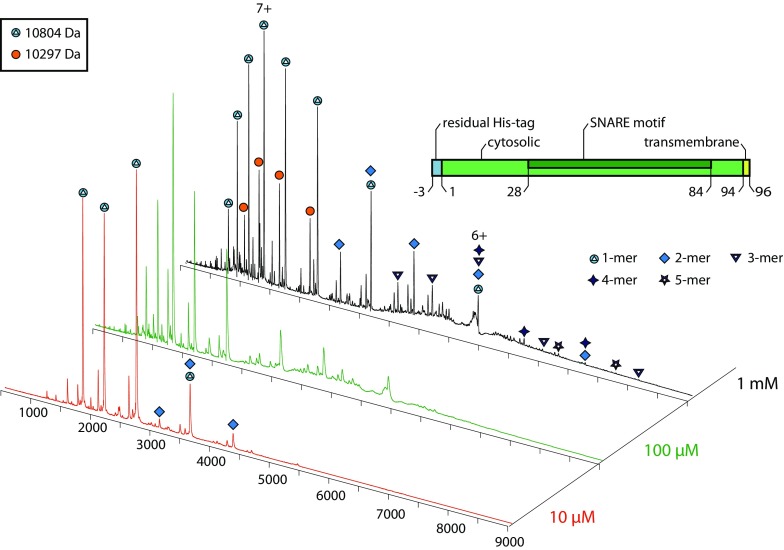
Oligomerisation of Syb(1-96) identified by native mass spectrometry. Mass spectra are shown for 10 μM, 100 μM and 1 mM Syb(1-96). Intact Syb(1-96) (blue circles; 10,804 Da) as well as a smaller fragment (orange circles; 10,297 Da) was observed. Dimeric Syb(1-96) is observed at 10 μM. Higher concentrations reveal higher oligomers (various symbols)

Next, we studied the intact protein by native mass spectrometry as described above. At a concentration of 10 μM, we observed one major charge state distribution corresponding to the monomeric protein (Figure 4). However, we also identified a second species corresponding to the mass of a Syb(1-96) dimer. To enhance signal intensity for the dimeric species as well as potential higher oligomers, we also used higher concentrations. Indeed, low abundant peak series corresponding to Syb(1-96) oligomers up to pentamers were identified in these solutions. Of these, the dimeric species is most abundant and higher oligomers appear at lower intensities. This pattern of Synaptobrevin-2 oligomers suggests that oligomerisation increases with concentration in an ‘aggregation-like’ manner rather than promoting the formation of defined oligomers.

Similar to full-length Synaptobrevin-2, we used chemical cross-linking to visualise these oligomers by gel electrophoresis and western blotting. As observed in the mass spectrum of intact Syb(1-96), we identified oligomers up to hexamers in the Coomassie-stained gel. Sensitive western blotting revealed oligomer formation at higher intensities (Figure S1 and Figure 3c). We then cut protein bands of cross-linked Syb(1-96) and analysed them as described (see ‘Methods’ for details). In total, we identified 43 cross-links between lysine, serine and threonine residues and 16 of these cross-links could be assigned to inter-protein interactions. Although the number of identified inter-molecular cross-links increased, the observed cross-linking pattern is similar to that obtained from cross-linking full-length Syb(1-116) showing that loss of the transmembrane domain did not change inter-protein interactions (Figure 3c). However, as shown by native mass spectrometry and cross-linking, formation of oligomers is enhanced when the transmembrane domain is deleted from the full-length protein.

### Increased Oligomerisation of C-Terminal Fragment Syb(49-96)

In the SNARE complex, Synaptobrevin-2 is part of a four-helix bundle [8]. To study whether structural elements of Synaptobrevin-2 are involved in oligomer formation, we next employed a C-terminal fragment of the cytosolic domain, namely Syb(49-96), containing the C-terminal half of the SNARE motif as well as the juxtamembrane domain. A previous study employing full-length and cytosolic Synaptobrevin-2 showed that it is natively disordered and, in the presence of dodecylphosphocholine micelles, forms helical segments (residues 36-54 and residues 77-88) [14] which are partially included in the fragment used here. In addition, a coiled coil trigger site important for SNARE complex formation is preceding the sequence of Syb(49-96) (residues 42-48) [39] but is missing in this variant. Syb(49-96) therefore represents a variant which is at the interface of structured and disordered Synaptobrevin-2 and likely provides insights into the importance of structural elements for Synaptobrevin-2 oligomerisation.

**Figure 5:**
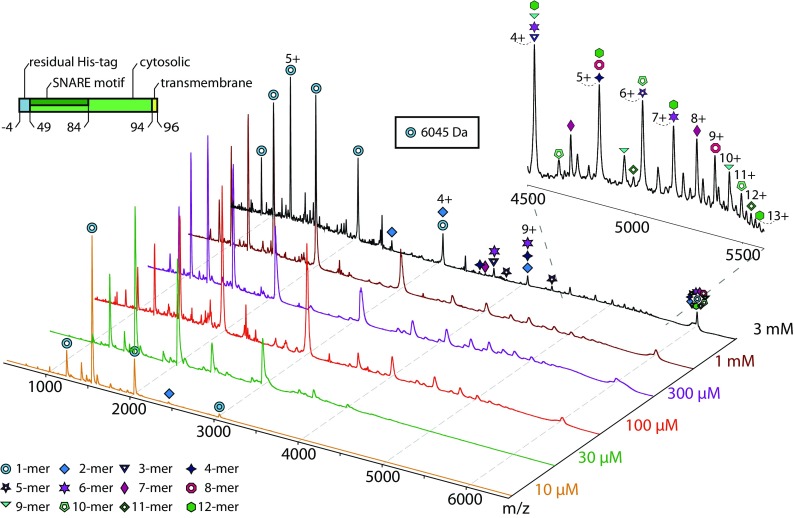
Oligomerisation of Syb(49-96) identified by native mass spectrometry. Mass spectra are shown for 10 μM, 30 μM, 100 μM, 300 μM, 1 mM and 3 mM Syb(49-96). Intact Syb(49–96) (blue circles; 6045 Da) as well as dimeric Syb(49–96) was observed at 10 μM. Higher concentrations reveal higher oligomers (various symbols)

First, we purified the protein as described (see ‘Methods’ for details) and confirmed the sequence after tryptic digestion (Figure 1). As described above, we then studied Syb(49-96) at varying concentrations. Native mass spectrometry revealed monomeric Syb(49-96) and additional oligomers with increasing concentration (Fig. 5). When compared with Syb(1-96), this truncated version formed higher oligomers; close inspection of the *m/z* region between 4000 and 6000 revealed the presence of up to dodecamers (Fig. 5, insert). The peak at approx. 6000 *m/z* corresponds to the mass of Syb(49-96) and therefore contributes to all charge state series of all oligomers present. Other peaks, for instance at 3000 or 4000 *m/z*, also contribute to more than one charge state series yielding the typical pattern for additive oligomer formation (see above).

In agreement with the results obtained from native mass spectrometry, chemical cross-linking also revealed the presence of higher oligomers as visualised by gel electrophoresis and western blotting. Again, oligomers up to decamers were observed (Figure S1 and Figure 3d). We then cut the protein bands of cross-linked Syb(49-96) oligomers and analysed them as described. Even though this Synaptobrevin-2 variant has the shortest sequence, we identified a relatively high number of cross-links, i.e. 33 cross-links compared with 43 and 23 cross-links in Syb(1-96) and Syb(1-116), respectively. Of these 33 protein interactions, 5 cross-links between lysine and serine or threonine as well as 17 inter-molecular cross-links were obtained after validation of the mass spectra. In fact, every lysine, serine and threonine residue in the Syb(49-96) sequence was found to be cross-linked in these experiments. Accordingly, a dense interaction network was obtained for Syb(49-96) oligomers (Figure 3d).

### Ion Mobility Mass Spectrometry of Syb(49-96) Reveals a Polydisperse Mixture

The presence of higher Syb(49-96) oligomers when compared with full-length or cytosolic Synaptobrevin-2 suggests that this particular fragment is prone to oligomerisation or even aggregation. This might be due to intrinsically disordered regions present in the C-terminal fragment used here. Ion mobility combined with mass spectrometry is ideally suited for structural analysis of proteins and yields information about conformational dynamics. In an ion mobility experiment, ions are guided by a weak electric field through a gas-filled drift tube. During this migration, extended ions undergo more collisions with the buffer gas than compact ions and, as a result, they are retained. The result is a separation of ions beyond simple *m/z* but also based on differences in size and shape [40]. We employed a home-built drift-tube ion mobility mass spectrometer [33] and analysed Syb(49-96) as described (see ‘Methods’ for details).

**Figure 6:**
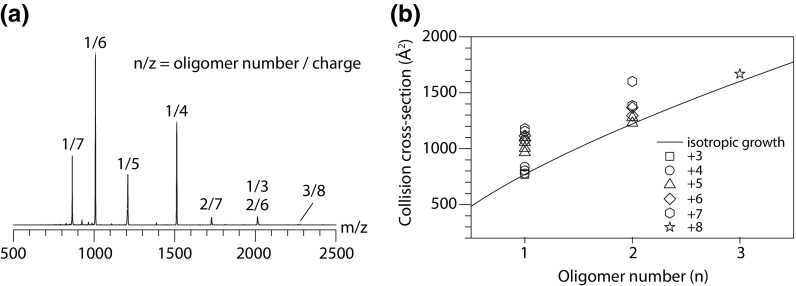
Ion mobility mass spectrometry of Syb(49-96). **(a)** Mass spectrum obtained with the home-built drift-tube mass spectrometer [33]. Peaks are labelled according to the oligomer and charge (*z*). **(b) **CCSs for each oligomer determined from ATDs

Due to the limited transmission for high *m/z* species of the home-built drift-tube mass spectrometer, we, however, only observed mass peaks that correspond to charge states for the lower oligomers, i.e. monomeric, dimeric and one charge state for trimeric Syb(49-96) (Figure 6a). The mass peaks are labelled with their *n/z* ratio, where *n* stands for the number of Syb(49-96) units within the cluster and *z* is the charge. Almost all species exhibit a broad ATD (Figure S5) which highlights the polymorphic character of this variant, i.e. more than one conformation is present for one and the same oligomeric state. While for the triply charged monomer *n/z* = 3/1, a narrow drift time peak is observed, higher monomer charge states (4+ to 7+) show the presence of up to four individual conformations. Dimers exhibit at least two distinct folds (6+ and 7+), whereas the trimer (*n/z* = 3/8) ATD only shows one narrow feature.

For all species, the respective CCS (Ω) was calculated (‘Methods’). The CCS is a molecular property that represents the overall shape of an ion and therefore allows to derive information about the assembly pathway. Figure 6b shows the experimental CCS values as a function of the oligomeric state *n*. The solid line represents the theoretical isotropic growth [41], i.e. growth for an idealised spherical assembly, following the equation Ω = σ¬1∙*n*2/3, where σ¬1 is the monomers CCS and *n* is the oligomer number. Species that exhibit CCS values close to the theoretical isotropic growth are most likely unstructured, whereas more extended conformations can be partially structured (helical or β-sheet-rich) or might result from Coulomb-induced unfolding. We found that the lower charge states of Syb(49-96) oligomers, which most likely represent the native solution structures, agree well with the isotropic growth and therefore likely represent unstructured and globular assemblies. For the higher charge states, on the other hand, significantly higher CCSs were observed. The presence of extended conformations for these species is, however, most likely a result of Coulomb repulsion due to a high charge density within the cluster rather than the formation of a structured oligomer.

## Discussion

Dimerisation, and in some cases oligomerisation, of Synaptobrevin-2 was controversially discussed in previous studies [15, 16, 18, 19]. To date, oligomerisation of Synaptobrevin-2 was mostly considered to be driven by the transmembrane domain. Here, we employed the full-length proteins as well as variants of the cytosolic domain and found that oligomerisation occurs in all variants in solution including those which are missing the transmembrane domain. Using chemical cross-linking, we consequently identified numerous interactions in the cytosolic domain. Our experiments therefore suggest that Synaptobrevin-2 oligomerisation is not restricted to interactions between the membrane domains but also includes interactions in the soluble part of the protein.

Surprisingly, when reconstituting full-length Synaptobrevin-2 into the lipid bilayer of liposomes, we did not observe oligomer formation. This might be due to separation of the monomers in the lipid bilayer as a consequence of a rather high lipid:protein ratio (i.e. 300:1 lipid:protein corresponding to 20 copies of Synaptobrevin-2 per liposomes compared with 70 copies Synaptobrevin-2 in synaptic vesicles [3]). Nonetheless, the absence of Synaptobrevin-2 oligomers in a native-like environment suggests that oligomerisation is not predominantly driven by the transmembrane domain or might require the presence of a specific lipid environment as reported for other proteins (e.g. Syntaxin-1A [42–46]). As the lipid mixture used here resembles the lipid content in native synaptic vesicles [3] and was proven to promote membrane fusion in vitro [37], it should provide a suitable lipid bilayer suggesting that Synaptobrevin-2 oligomerisation does not depend on the lipid environment. The absence of oligomers in proteoliposomes therefore suggests that oligomer formation is rather caused by disordered regions in the cytosolic domain of the protein.

Considering the size of the Synaptobrevin-2 variants used here, and taking the number of lysine as well as serine, threonine and tyrosine residues into account, the number of observed cross-links is impressive. This agrees well with previous studies which showed that the cytosolic domain is natively unstructured and highly flexible in the absence of lipids or other SNARE proteins [6, 9, 14] therefore allowing formation of numerous cross-links in the highly flexible cytosolic domain. Although the functionally active SNARE complex is a highly structured four-helix bundle [7, 8], flexibility of the cytosolic domain appears to be important for spontaneous formation of the SNARE complex on demand. Incorporation into the lipid bilayer as performed here might induce formation of helical fragments described earlier [14] and might also explain the absence of oligomers.

Comparing the different Synaptobrevin-2 variants in solution, we found that the degree of oligomerisation increased in the truncated versions. This is particularly true for the mass spectra of non-covalently linked oligomers, while we could not preserve oligomers of full-length Syb(1-116), pentamers and even dodecamers were observed for Syb(1-96) and Syb(49-96), respectively. This is surprising considering previous studies which described tight interactions formed by the transmembrane domain [17]. When comparing the full-length or the intact cytosolic domain with the shorter Syb(49-96) variant, the latter is characterised by a missing coiled coil trigger site [39] and helical segments [14] which might cause development of structural elements in the longer variants. We therefore propose that Syb(49-96) is natively disordered and therefore is prone to oligomerisation. Indeed, by applying ion mobility mass spectrometry, we found that lower charge states of monomers, dimers and trimers, which likely represent solution structures, follow an isotropic growth curve which is typical for formation of soluble oligomers of intrinsically disordered proteins [41]. This is supported by an impressive number of intra- and inter-molecular cross-links in this short variant.

Even though a high number of cross-links was determined in this study, all identified cross-links are located in the cytosolic domain. Inspection of the amino acid sequence of the transmembrane domain reveals that no lysine, serine and threonine residues are present. There is only one tyrosine residue (Y113) present; however, cross-links including tyrosine were not observed in our experiments and are likely missing here too. The two cross-linkable residues at the C-terminus of the transmembrane domain (S115 and T116) should be accessible to the BS3 cross-linker [35]; however, we did not observe spectra including the C-terminus of Synaptobrevin-2. Possible reasons might be the, to some extent, restricted access of the cross-linking reagent due to the detergent micelle as well as limited proteolysis in the membrane domain (see Figure 1) which is making the analysis of cross-linked dipeptides in this segment difficult. The cross-linking strategy followed here might therefore not be ideal to study protein interactions in the transmembrane domain alone and we therefore employed the full-length protein. Nonetheless, oligomerisation of Synaptobrevin-2 could be studied and the combination of native mass spectrometry and chemical cross-linking allowed distinguishing between the three variants. Removal of the membrane domain increased cross-linking in solution as determined in the mass spectra of the intact assemblies as well as by gel electrophoresis and western blotting.

From a technical point of view, the need for manual validation of cross-linking data was recently discussed [47]. Here, we validated the spectra of potential cross-linked dipeptides according to the following criteria: (i) fragment ion series (y- or b-ions) must be observed for both peptides; (ii) we only considered peptides with at least four amino acids; (iii) the major peaks of the spectra should be assigned; (iv) assigned peaks should have a certain intensity (i.e. should be well above the signal-to-noise). To overcome the uncertainty of isobaric cross-links originating from consecutive peptide sequences [47], we further checked the list of potential cross-links for consecutive peptides and, in the case these were obtained, for characteristic ions confirming the presence of cross-linked dipeptides. Nonetheless, in the case of wrongly assigned isobaric cross-links, these would only be considered as intra-molecular interactions and therefore would not change the outcome of this study. In the protein assemblies studied here as well as in other protein complexes, intra-molecular cross-links do not affect the generation of structural models and rather provide information on solvent accessibility of the protein assembly. The latter would be the same in the case of an assigned intra-molecular or ‘dead-end’ cross-link.

Considering the size and the number of lysine residues of the Synaptobrevin-2 variants used in this study, the number of obtained cross-links is relatively high (see Table S2). We identified many cross-links within monomeric Synaptobrevin-2 as well as cross-links that could unambiguously be assigned to inter-protein interactions of the monomeric subunits. The sequences of these cross-linked dipeptides were exactly the same or contained overlapping sequence stretches and, therefore, they must be generated from two copies of Synaptobrevin-2 as the protein sequence does not contain repeating peptide sequences (see Figure S2 for an example spectrum). Together with the mass of the intact dipeptide, they unambiguously represent inter-protein cross-links of two Synaptobrevin-2 monomers. This is of particular importance as inter-protein interactions in homo-oligomers are usually difficult to identify by cross-linking and in most cases require labelling with stable isotopes, for instance ^15^N, including specialised data analysis workflows [48]. The identification of inter-protein interactions in homo-oligomers without stable isotope labelling, as observed here, is only possible if the monomeric subunits align in a parallel (see refs [49, 50] for examples) or assemble in a globular, unstructured manner. Based on the ion mobility experiments as well as the observed cross-linking pattern, and the fact that Synaptobrevin-2 has been shown to be unstructured in the absence of its SNARE complex interaction partners SNAP25 and Syntaxin-1A, we conclude that the latter scenario is the case.

## Conclusion

In previous studies, native mass spectrometry was successfully combined with cross-linking mainly targeting multi-subunit protein complexes [51–53]. Here, we show that this combination is also beneficial for the structural analysis of homo-oligomers, which is challenging for the individual techniques. Using native mass spectrometry allows determining the oligomeric states of the assemblies as well as their intensities and therefore provides insights into the type of oligomerisation. Combined with ion mobility, the conformational distribution of the assemblies and formation of secondary structures is revealed [41, 54]. This is of particular importance when distinguishing between formation of ordered (e.g. fibrils) and disordered (e.g. aggregates) assemblies. In this study, we found that oligomerisation is increasing with concentration and oligomers are formed in an additive (‘aggregation-like’) manner rather than oligomers of defined stoichiometry.

Cross-linking protein oligomers, on the other hand, unravels their interaction sites including those between different domains and in some cases the monomeric building blocks. Gel electrophoresis and western blotting, as shown here, allow estimating the degree of oligomerisation. Importantly, in some cases, cross-linking can capture transient or low abundant protein interactions when the application of native mass spectrometry is technically limited. Considering the protein interactions identified here between the monomeric building blocks of the oligomers (i.e. inter-protein cross-links), we do not identify interactions between defined stretches of Synaptobrevin-2 variants but rather a random arrangement highlighting again the formation of non-ordered oligomers. Of note, cross-linking only reveals binary protein interactions; that means the cross-links identified in one experiment represent a mixed population of interactions between various monomeric building blocks. In general, the combination of both techniques is beneficial for the structural analysis of homo-oligomers including the oligomeric states and protein interactions and, in future studies, paves the way for structural modelling.

## Electronic Supplementary Material

ESM 1 (DOCX 746 kb)
