# Supplementary material for: Oligomerisation of Synaptobrevin-2 Studied by Native Mass Spectrometry and Chemical Cross-Linking
Source: J Am Soc Mass Spectrom. 2018 Jun 12;30(1):149–60. doi: 10.1007/s13361-018-2000-4 (PMC6318248; doi:10.1007/s13361-018-2000-4)
Supplement: Supplementary file 1 — (DOCX 746 kb) [file 13361_2018_2000_MOESM1_ESM.docx]

Supplementary Material

Oligomerisation of Synaptobrevin-2 studied by native mass spectrometry and chemical cross-linking

Sabine Wittig^1^, Caroline Haupt^1^, Waldemar Hoffmann^2,3^, Susann Kostmann^1^, Kevin Pagel^2^, Carla Schmidt^1*^

^1^ Interdisciplinary research center HALOmem, Martin Luther University Halle-Wittenberg, Kurt-Mothes-Str. 3a, 06120 Halle (Saale), Germany.

^2^ Freie Universität Berlin, Institute of Chemistry and Biochemistry – Organic Chemistry, Takustr. 3, 14195 Berlin, Germany.

^3^ Fritz-Haber-Institut der Max-Planck-Gesellschaft, Faradaystr. 4-6, 14195 Berlin, Germany.

*** Correspondence:** Carla Schmidt, Interdisciplinary research center HALOmem, Institute for Biochemistry and Biotechnology, Martin Luther University Halle-Wittenberg, Kurt-Mothes-Str. 3a, 06120 Halle (Saale), Germany

**Figures**

**
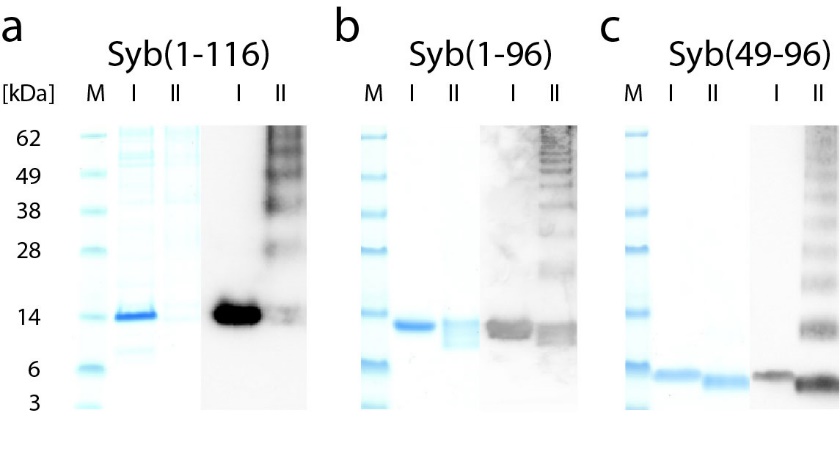
**

**Figure S1. Cross-linking of Synaptobrevin-2 variants.** 10 μM of each variant were cross-linked with 200 μM BS3. I: Synaptobrevin-2; II: Synaptobrevin-2 + BS3.

**a.** Syb(1-116). **b.** Syb(1-96). **c.** Syb(49-96).

**
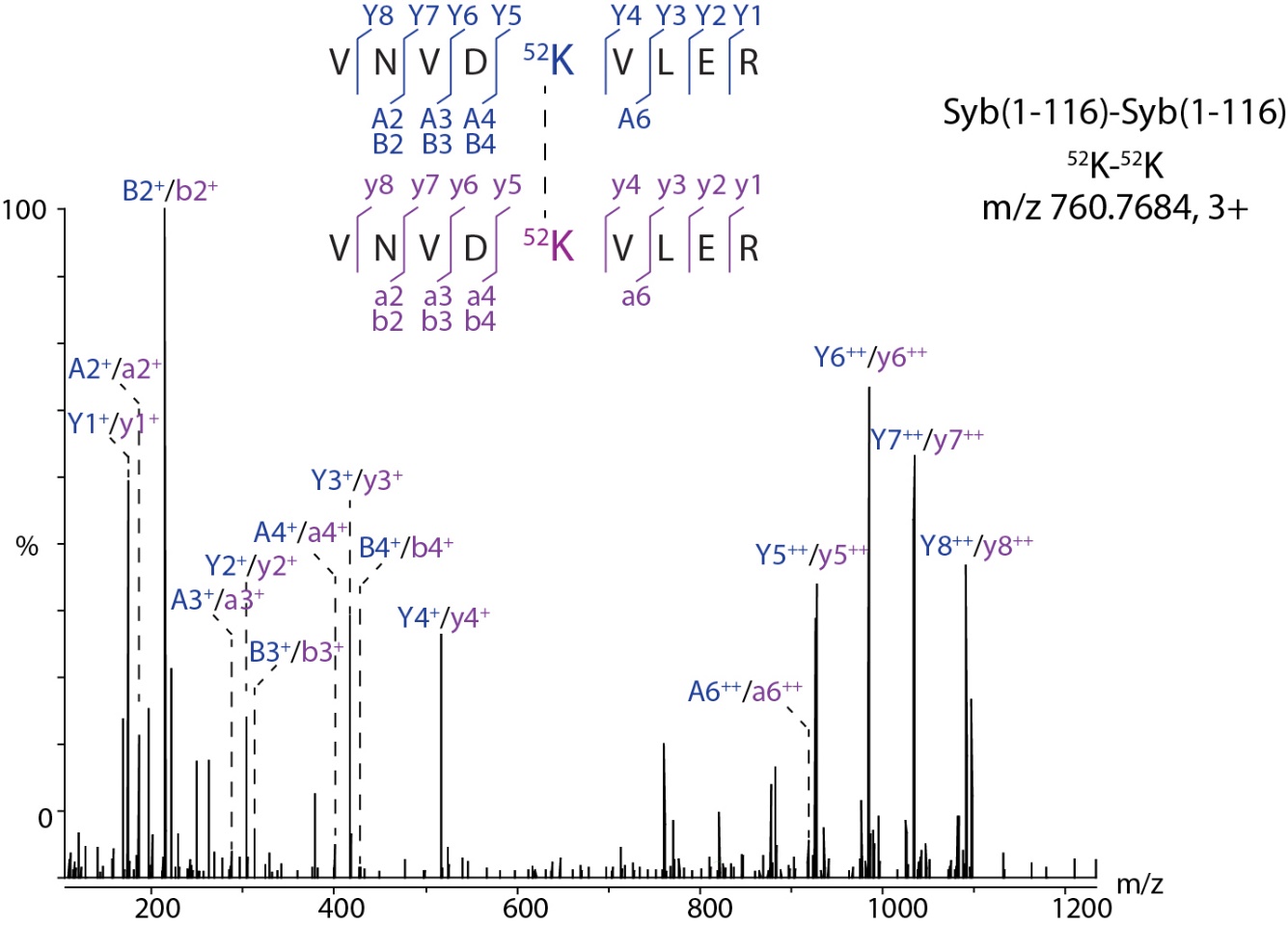
**

**Figure S2. Example spectrum of an inter-molecular cross-link.** Complete y-ion series as well as several b-ions were obtained.


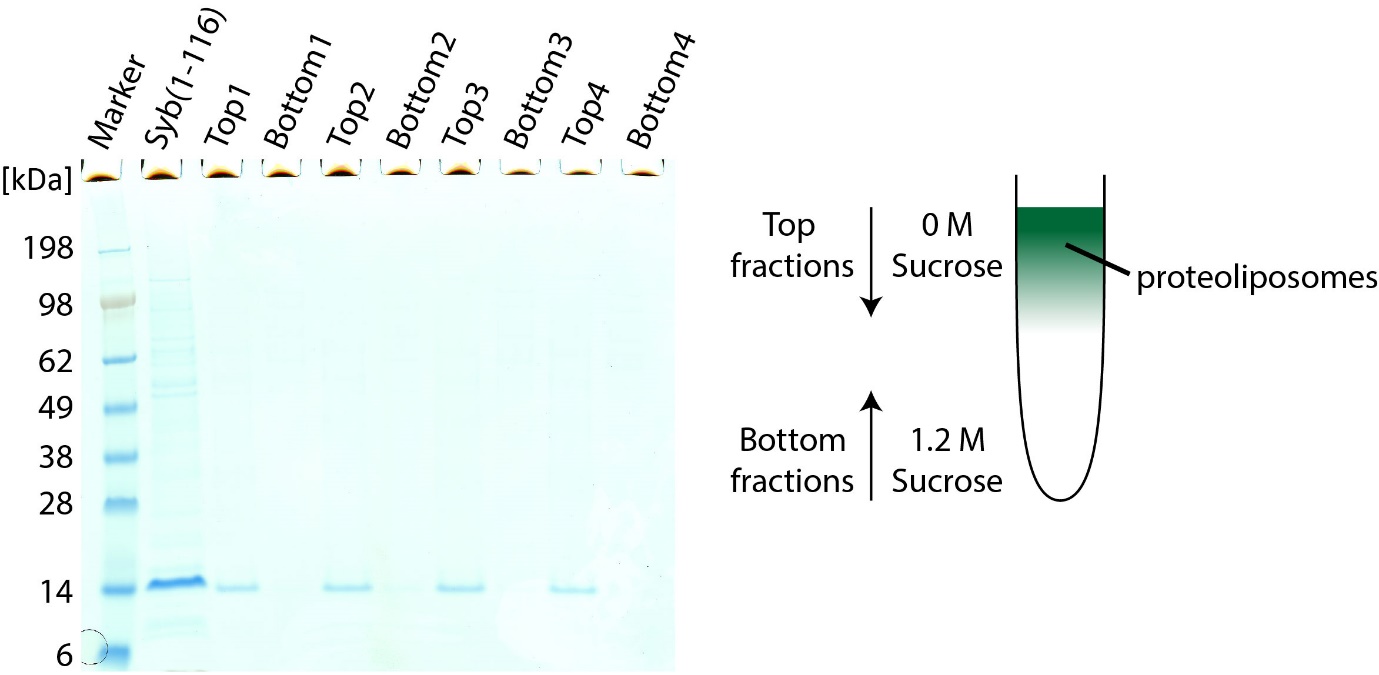


**Figure S3. Flotation assay of proteoliposomes.** Top and bottom fractions of the sucrose gradient (rhs) were evaluated by gel electrophoresis (lhs). Synaptobrevin-2 was observed in top fractions while bottom fractions were empty.


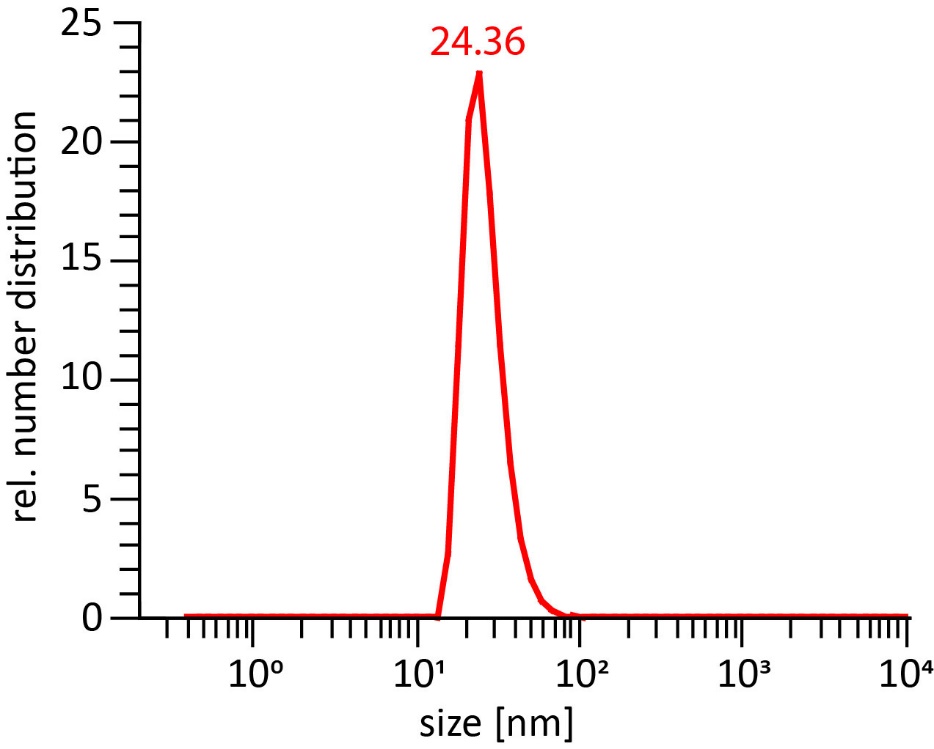


**Figure S3. Dynamic light scattering confirms a homogeneous population of proteoliposomes.** A mean diameter of approximately 24 nm was obtained.


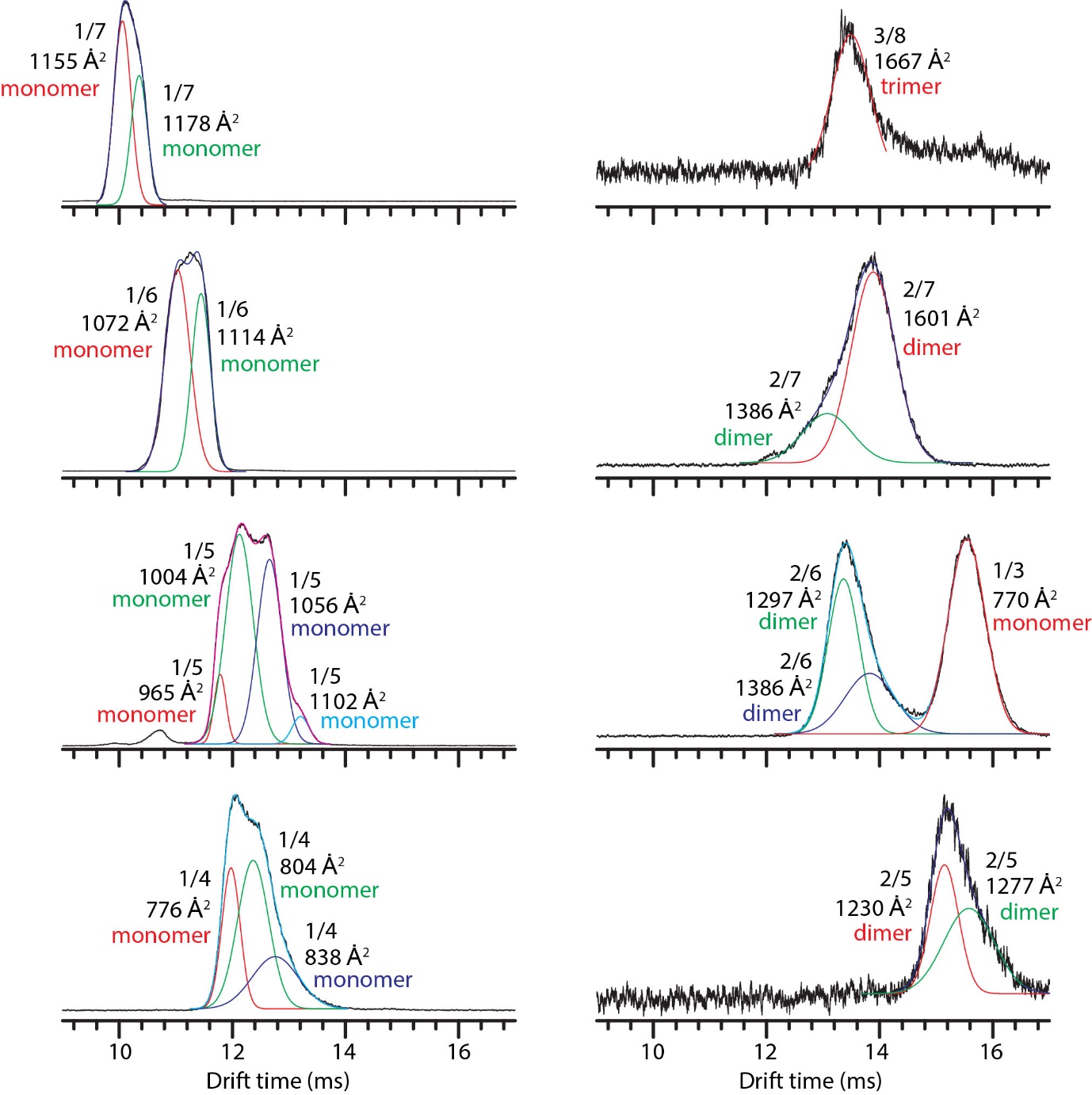


**Figure S5. Ion mobility-mass spectrometry of Syb(49-96).** The ATD is shown for each oligomeric state. More than one conformation is present for one and the same oligomeric state. The CCSs determined for each oligomer and charge state are shown. The oligomer number / charge (n/z) is given.

**Tables**

**Table S1: Masses of identified Synaptobrevin-2 oligomers.** Experimentally obtained and theoretical masses as well as the mass difference (Δm) are given for each oligomer.

| **Oligomer** | **m (exp) [Da]** | **m (theor) [Da]** | **Δm [Da]** |
| --- | --- | --- | --- |
| **Syb(1-116)** | | | |
| 1-mer | 12978 | 12972 | 6 |
| 1-mer fragment | 12481 |  |  |
| **Syb(1-96)** | | | |
| 1-mer | 10804 | 10799 | 5 |
| 1-mer fragment | 10297 |  |  |
| 2-mer | 21604 | 21598 | 6 |
| 3-mer | 32405 | 32398 | 7 |
| 4-mer | 43209 | 43197 | 12 |
| 5-mer | 54010 | 53996 | 14 |
| **Syb(1-49)** | | | |
| 1-mer | 6045 | 6040 | 5 |
| 1-mer fragment | 5540 |  |  |
| 2-mer | 12083 | 12080 | 3 |
| 3-mer | 18137 | 18120 | 17 |
| 4-mer | 24174 | 24160 | 14 |
| 5-mer | 30213 | 30200 | 14 |
| 6-mer | 36282 | 36239 | 43 |
| 7-mer | 42303 | 42279 | 24 |
| 8-mer | 48348 | 48319 | 29 |
| 9-mer | 54395 | 54359 | 36 |
| 10-mer | 60447 | 60399 | 48 |
| 11-mer | 66478 | 66439 | 39 |
| 12-mer | 72514 | 72479 | 35 |

**Table S2: Cross-links identified in Synaptobrevin-2 variants.** The two cross-linked residues are given for each identified and validated cross-link. Residues are numbered according to the full-length protein. For each variant, the highest score and the number of obtained MS/MS spectra is given for each identified cross-links. Cross-links that were unambiguously identified as inter-protein cross-link are shaded in grey. Of note, due to different peptide length after tryptic digestion and different length of the Synaptobrevin-2 variants some cross-links were assigned to be either inter- or intra-peptide cross-links.

|  |  | **Syb(49-96)** | | **Syb(1-96)** | | **Syb(1-116)** | |
| --- | --- | --- | --- | --- | --- | --- | --- |
| **Residue 1** | **Residue 2** | **Score** | **# Spectra** | **Score** | **# Spectra** | **Score** | **# Spectra** |
| 87 | 91 |  |  | 6.88E-09 | 1 |  |  |
| 85 | 91 |  |  | 2.47E-12 | 3 |  |  |
| 85 | 87 | 2.30E-10 | 3 | 5.20E-10 | 2 |  |  |
| 85 | 85 | 3.12E-02 | 1 |  |  |  |  |
| 83 | 91 | 6.17E-10 | 11 | 3.67E-11 | 15 |  |  |
| 83 | 87 | 8.98E-13 | 6 | 6.83E-14 | 20 |  |  |
| 83 | 83 | 6.01E-03 | 4 | 2.85E-06 | 12 | 2.13E-03 | 2 |
| 83 | 61 |  |  | 3.49E-08 | 4 | 4.97E-07 | 4 |
| 80 | 85 | 8.70E-04 | 4 |  |  |  |  |
| 80 | 83 | 8.27E-05 | 5 |  |  |  |  |
| 79 | 85 | 1.27E-03 | 7 |  |  | 3.64E-04 | 2 |
| 79 | 83 | 1.95E-05 | 11 | 1.14E-06 | 11 |  |  |
| 79 | 79 | 6.44E-05 | 8 | 4.32E-05 | 5 | 2.16E-04 | 4 |
| 75 | 85 | 1.78E-03 | 1 |  |  |  |  |
| 75 | 83 | 2.52E-03 | 1 |  |  |  |  |
| 61 | 61 | 2.48E-03 | 3 |  |  |  |  |
| 59 | 91 |  |  | 8.70E-13 | 6 |  |  |
| 59 | 87 |  |  | 1.96E-08 | 16 |  |  |
| 59 | 83 |  |  | 3.27E-16 | 12 | 1.50E-15 | 16 |
| 59 | 80 |  |  | 9.57E-06 | 1 | 1.88E-07 | 4 |
| 59 | 79 | 2.60E-06 | 9 | 4.33E-05 | 1 | 1.33E-10 | 10 |
| 59 | 75 | 1.23E-06 | 2 |  |  |  |  |
| 59 | 61 | 7.01E-04 | 1 |  |  |  |  |
| 59 | 59 | 1.49E-07 | 5 | 2.04E-10 | 4 |  |  |
| 52 | 91 |  |  | 3.17E-19 | 6 |  |  |
| 52 | 87 |  |  | 8.43E-13 | 11 |  |  |
| 52 | 83 |  |  | 1.96E-13 | 14 | 4.54E-15 | 32 |
| 52 | 80 |  |  |  |  | 3.66E-11 | 13 |
| 52 | 79 |  |  |  |  | 5.03E-11 | 14 |
| 52 | 61 |  |  |  |  | 1.54E-11 | 13 |
| 52 | 59 |  |  | 2.96E-18 | 18 | 2.73E-19 | 87 |
| 52 | 52 |  |  | 2.13E-11 | 15 | 7.10E-10 | 13 |
| 35 | 85 |  |  | 1.52E-11 | 2 | 1.31E-15 | 5 |
| 35 | 35 |  |  |  |  | 5.92E-05 | 1 |
| 7 | 80 |  |  |  |  | 2.02E-08 | 5 |
| 7 | 61 |  |  |  |  | 2.25E-10 | 8 |
| 7 | 7 |  |  | 7.53E-15 | 193 | 3.10E-02 | 5 |
| 4 | 85 |  |  |  |  | 1.29E-08 | 2 |
| 4 | 83 |  |  | 2.42E-08 | 13 | 4.05E-09 | 6 |
| 4 | 4 |  |  | 6.07E-12 | 15 |  |  |
| 2 | 85 |  |  | 4.65E-10 | 3 |  |  |
| 2 | 79 |  |  | 9.02E-08 | 3 |  |  |
| 2 | 59 |  |  | 1.50E-11 | 14 | 1.38E-09 | 9 |
| 2 | 7 |  |  | 2.78E-18 | 33 |  |  |
| 2 | 4 |  |  | 6.05E-17 | 6 |  |  |
| 2 | 2 |  |  | 1.15E-24 | 32 |  |  |
| N-Ser | 91 |  |  | 2.84E-06 | 24 |  |  |
| N-Ser | 87 | 7.36E-04 | 3 | 9.01E-13 | 32 |  |  |
| N-Ser | 85 |  |  | 2.57E-09 | 8 |  |  |
| N-Ser | 83 | 2.76E-11 | 42 |  |  |  |  |
| N-Ser | 79 | 5.57E-06 | 21 |  |  |  |  |
| N-Ser | 61 | 7.32E-05 | 6 |  |  |  |  |
| N-Ser | 59 | 8.02E-15 | 57 | 5.59E-16 | 51 | 6.93E-14 | 23 |
| N-Ser | 52 | 2.25E-11 | 19 |  |  | 2.33E-08 | 40 |
| N-Ser | 7 |  |  | 1.34E-17 | 20 |  |  |
| N-Ser | 4 |  |  | 3.81E-25 | 21 |  |  |
| N-Ser | 2 |  |  | 4.08E-24 | 29 |  |  |
| N-Ser | N-Ser | 2.61E-14 | 259 | 5.86E-25 | 131 |  |  |
| N-Term | 94 | 2.55E-06 | 30 |  |  |  |  |
| N-Term | 87 | 6.51E-13 | 5 |  |  |  |  |
| N-Term | 85 |  |  | 6.11E-09 | 2 |  |  |
| N-Term | 83 | 4.67E-10 | 7 |  |  |  |  |
| N-Term | 80 | 2.37E-12 | 1 |  |  |  |  |
| N-Term | 61 | 5.93E-11 | 14 |  |  |  |  |
| N-Term | 59 | 2.36E-15 | 55 | 3.22E-09 | 5 |  |  |
| N-Term | 52 | 5.84E-11 | 24 |  |  |  |  |
| N-Term | 7 |  |  | 3.90E-08 | 3 |  |  |
| N-Term | 2 |  |  | 9.49E-15 | 5 |  |  |
| N-Term | N-Ser | 9.35E-11 | 30 |  |  |  |  |
| N-Term | N-Term | 1.07E-18 | 377 | 1.01E-16 | 16 |  |  |
